# Applying the Social-Ecological Framework to Link the Drivers of Intimate Partner Violence Among Women in the Caribbean and Their Risk for HIV Infection

**DOI:** 10.7759/cureus.49427

**Published:** 2023-11-26

**Authors:** Nyla Lyons, Brendon Bhagwandeen

**Affiliations:** 1 Research, Medical Research Foundation, Port of Spain, TTO; 2 Mathematical and Computer Sciences, Heriot-Watt University Malaysia, Putrajaya, MYS

**Keywords:** gbv prevention, social -ecological framework, women, hiv prevention, caribbean, gender-based violence

## Abstract

For countries with a high prevalence of HIV such as in the Caribbean, intimate partner violence (IPV) may increase the chances for acquiring HIV infection. Using secondary data, we compared findings from studies conducted in five Caribbean countries measuring the prevalence of gender-based violence among women in Grenada, Jamaica, Guyana, Suriname, and Trinidad and Tobago. The Social-Ecological Framework was used to categorize women's dual risk for intimate partner violence and HIV. We found that younger age, lower education, childhood experiences of abuse, income dependency, controlling behaviors of partners, non-disclosure of violence, and early sexual experiences were associated with intimate partner violence. These factors also predispose women in the Caribbean to HIV infection. The Social-Ecological Framework is applicable to understanding the drivers of intimate partner violence and HIV infection at multiple levels and for the design and promotion of combined prevention interventions. Our study also demonstrated the applicability of the Social-Ecological Framework as an analytical and predictive model underscoring the need for increased coordination across multiple actors to strengthen advocacy, given the pervasiveness of harmful social norms and gender inequalities which undermine IPV and HIV control efforts.

## Introduction

Gender-based violence (GBV) is a global, public health problem that can undermine AIDS control efforts. Studies investigating the intersection between violence against women (VAW) and HIV infection have been well established. Reviews on this intersection demonstrated the complexity of the relationship between HIV risk, violence, and context-specific gender norms [1].

In a systemic review of 32 studies in the United States, it was found that women who experience violence were at a greater risk for HIV infection [2]. Further, in a number of population-based studies a positive association was reported between different forms of violence and HIV infection to include sex workers and alcohol abusers [3,4]. Studies conducted in Kenya, Tanzania, and Southern Africa found that women’s risk for HIV infection increased threefold when they experienced intimate partner violence (IPV) compared to women who have not [5,6]. In countries with a high HIV prevalence, it was also documented that women who experience violence are 50% more likely to contract HIV [7,8].

The Joint United Nations Program of HIV/AIDS (UNAIDS, 2018) estimates that 30% of women globally have experienced physical and/or sexual intimate partner violence or sexual violence from a non-partner. Between 2016 and 2018, five studies conducted in Grenada, Guyana, Jamaica, Suriname and Trinidad and Tobago, measuring the point prevalence of gender-based violence, showed that on average, 46% of women experienced at least one form of violence in their lifetime. Lifetime prevalence rates of violence for women reporting any type of IPV ranged from 39% in Grenada and Trinidad and Tobago to 55% in Guyana. Across the five countries surveyed, the most prevalent form of IPV was emotional violence, and just over one-third of ever-partnered women aged 15-64 years, reported emotional violence across their lifetime [9]. Given the high prevalence in the region, it is critical to eliminate and prevent all forms of violence against women and girls by addressing the root causes and risk factors for which HIV is both a major cause and consequence. In the context of the Caribbean, studies linking gender-based violence and HIV have not been widely published. However, recent reports on the high prevalence of GBV in the region suggest that women experiencing violence are at increased risk for acquiring HIV. 

Using secondary data from studies conducted in five countries, this paper will apply the Social-Ecological Framework to characterize factors at the individual, interpersonal, institutional and societal levels associated with women’s risk for IPV, and by extension HIV infection. The use of the Social-Ecological Framework is further discussed as an analytical tool to understand the drivers of both HIV and intimate partner violence and to combine interventions accordingly.

## Materials and methods

The Social-Ecological Framework

**Figure 1:**
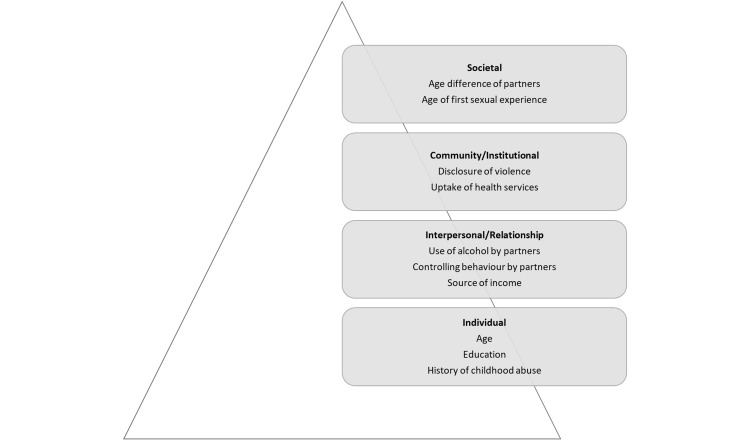
The Social-Ecological Framework

We used the Social-Ecological Framework as shown in Figure 1 to organize the individual, interpersonal, institutional, and societal factors linked to IPV, and discussed how these intersect with women’s risks for HIV infection. Bronfenbrenner’s Social-Ecological Framework [10,11] was expanded by McLeroy and colleagues [12] and used to explore how intrapersonal/individual factors, interpersonal processes, institutional/community factors, as well as societal/policies potentially impact the uptake of health interventions. The four-level Social-Ecological Model has been used to better understand the range of factors that put people at risk for violence or protect them from experiencing or perpetrating violence, and the effect of potential prevention strategies. The model has since been applied to qualitative research linking Social-Ecological factors influencing sexual risk-taking, HIV-related sexual resilience and risk-taking behaviors among young persons and with further implications for the uptake of HIV prevention interventions [13,14].

Individual Factors

The first level of the Social-Ecological Framework, identified the individual characteristics and personal history factors that increase the likelihood of becoming a victim or perpetrator of GBV. In the context of this paper, these included factors such as educational level, age, and history of childhood abuse reported by women subjected to any type of IPV across their lifetime.

Interpersonal/Relationship Factors

The second level of the Social-Ecological Framework, i.e., interpersonal factors, included factors that increased the risk of GBV against women because of relationships with peers, intimate partners, and family members. A person’s closest social circle, i.e., peers, and family members, can act as protective and/or risk factors for gender-based violence. In the context of this narrative, interpersonal factors included the use of alcohol by partners, women’s reported source of income, and controlling behaviors by partners. Controlling behavior by partners was defined as at least one affirmative response about specific behaviors exhibited by their partner including whether partners became angry or jealous when the woman spoke to another man, whether partners insisted on always knowing where the woman was, whether partners permitted the woman to meet with friends, and whether partners trusted the woman with money.

Institutional Factors

The third level of the Social-Ecological Framework, i.e., institutional/community factors, underscored settings such as social organizations and institutions to include healthcare settings, schools, workplaces, and neighborhoods, in which social relationships occurred and sought to identify the characteristics of these settings that were associated with becoming victims or perpetrators of GBV. In this context, these factors included disclosure of violence, and the uptake of health services as reported by women subjected to any type of IPV across their lifetimes.

Societal/Policy Factors

The fourth level of the Social-Ecological Framework viewed broad societal/policy factors that helped to create a climate in which GBV was perpetuated. These aspects included social equalities and cultural norms within a society and/or health, economic, educational, and social policy factors. In this paper, these factors included the age difference between partners, and age of first sexual experiences as reported by women subjected to any type of IPV across their lifetimes.

Data source

Secondary data were pooled data from five national prevalence surveys conducted between 2016 and 2018 in Grenada, Guyana, Jamaica, Suriname, and Trinidad and Tobago, measuring gender-based violence against women and the associated risk factors. The studies were conducted with the support of Caribbean Governments, UN Women, the United Nations Development Program (UNDP), the United States Agency for International Development (USAID), the Caribbean Development Bank (CDB), and the Inter-American Development Bank (IDB). The findings and summary data were made available to the public. In the countries surveyed, the study designs were similar and were adapted from the methodological approach and questionnaire developed by WHO to collect prevalence data on violence against women. Also, the approach used to collect and summarize the study data was consistent across all five countries, allowing for a comparison across countries to be facilitated. The studies each identified several factors significantly linked to gender-based violence, including individual characteristics of women, partner attributes, and women’s attitudes towards gender norms and roles (United Nations Entity for Gender Equality and the Empowerment of Women, 2021). For this paper, data on perceived factors across the five countries were pooled and then compared to describe central tendency values across the five countries. In this paper, factors linked to the point prevalence of lifetime intimate violence were arranged using the Social-Ecological Framework, where lifetime IPV has been defined as the proportion of ever-partnered women who reported that they were subjected to one or more acts of physical and/or sexual violence in their lifetime.

## Results

This section presented the results of our comparisons of the factors linked to women subjected to lifetime and current IPV in Grenada, Guyana, Jamaica, Suriname, and Trinidad and Tobago. These factors included education level, age, sources of income, history of childhood abuse, age difference of partners, disclosure of violence, partner use of alcohol, controlling behaviors by partners, health service uptake, and age of first sexual experience.

Individual factors of the Social-Ecological Framework: country comparisons of age, education, experiences of childhood abuse

**Figure 2:**
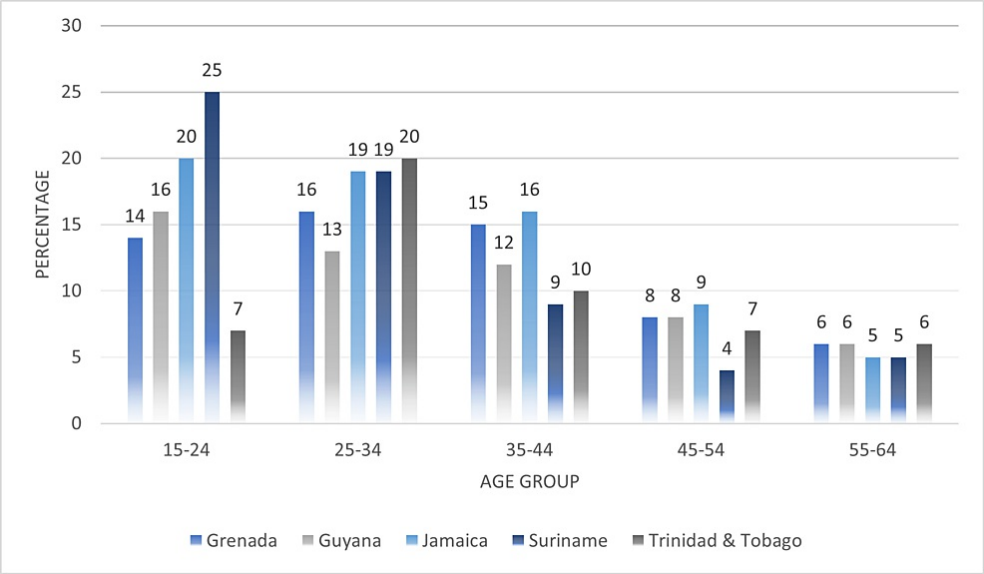
Comparing sources of disclosure among women who experience any type of intimate partner violence (IPV) across their lifetime

In Figure 2, we compared the age groups among women who were subjected to any type of IPV. It was reported that women aged 15-24 and 25-34 years showed the highest average point prevalence values of any type of IPV experienced in their lifetime (15-24 years: 16.4%, 25-34 years: 17.4%, 35-44 years: 12.4%, 45-54 years: 7.2%, 55-64 years: 5.6%). For women aged 15-24 years, Suriname reported the highest point prevalence for any form of IPV (25%). Trinidad and Tobago (20%), Suriname (19%) and Jamaica (19%) reported the highest proportions of women aged 25-34 years who experienced some form of IPV in their lifetime. It was reported that as the women grew older, the point prevalence of IPV experienced reduced.

**Figure 3:**
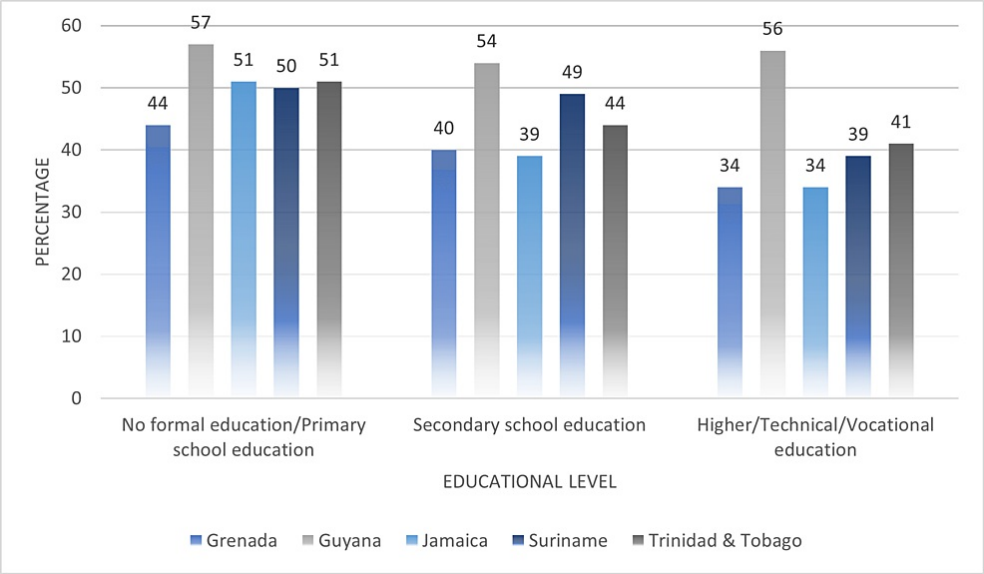
Comparing educational levels of women who experienced any type of intimate partner violence (IPV) across their lifetime

In Figure 3, we compared the educational levels of women who were subjected to any type of IPV. It was observed that women having either no formal education or up to primary school education displayed the highest average point prevalence of any type of IPV experienced in their lifetime (up to primary education: 50.6%, secondary education: 45.2%, higher education: 40.8%). Apart from Grenada (44%), the other four Caribbean countries presented proportions of 50% and above for women with up to primary school education and being subjected to any type of IPV. Across education levels, Guyana reported accounts of women experiencing some form of IPV as over 50% regardless of their educational level. The other four countries presented gradual reductions in the point prevalence of any type of IPV women experienced, as their education level increased.

**Figure 4:**
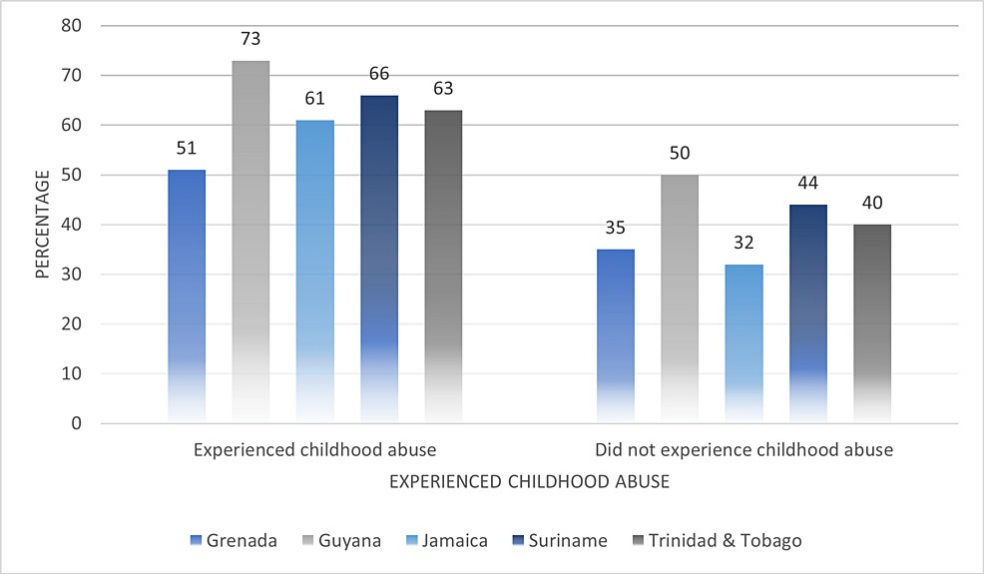
Comparing reports of childhood abuse among women who experienced any type of intimate partner violence (IPV) across their lifetime

In Figure 4, we compared reports of childhood abuse among women who were subjected to any type of IPV. On average, there was a higher point prevalence of women who experienced IPV given that they were subjected to abuse during their childhood (experienced childhood abuse: 62.8%, did not experience childhood abuse: 40.2%). Each of the five Caribbean countries reported proportions of women experiencing childhood abuse and some form of IPV that were over 50%, with Guyana having the highest point prevalence (73%).

**Figure 5:**
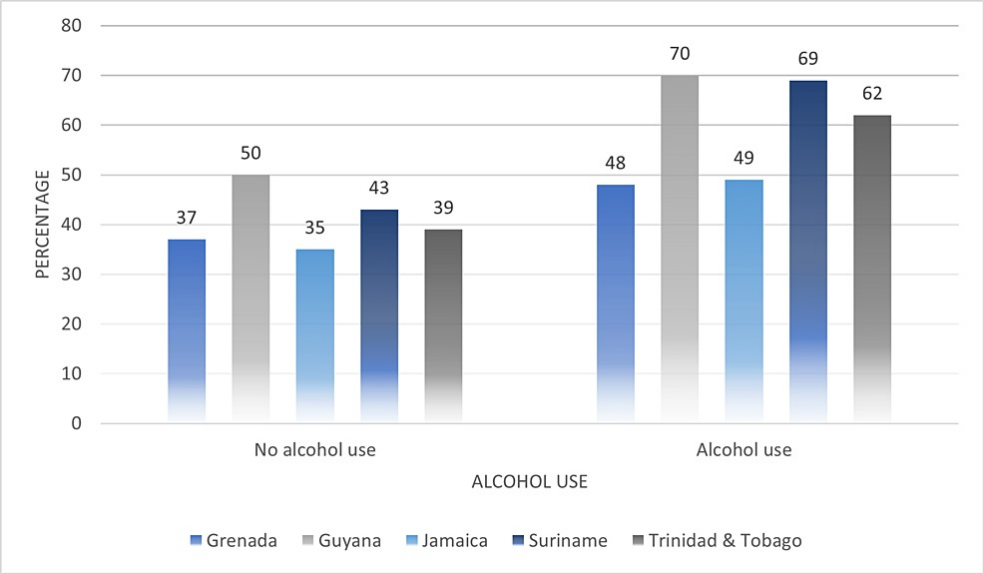
Comparing use of alcohol (at least once per week) by partners of women who experienced any type of intimate partner violence (IPV) across their lifetime

In Figure 5, we compared reports of alcohol use by partners among women who were subjected to any type of IPV. It was observed that women with partners who used alcohol at least once per week displayed the highest average prevalence values of any type of IPV experienced in their lifetime (no alcohol use: 40.8%, alcohol use: 59.6%). In Guyana, proportions of women experiencing any form of IPV given that their partner used alcohol were the highest (70%). Suriname also reported comparable proportions of women who experienced IPV given that their partners used alcohol at least once per week (69%). This was followed by the point prevalence reported in Trinidad and Tobago (62%).

Interpersonal factors of the Social-Ecological Framework: country comparisons of alcohol use by partners, source of income, controlling behaviors of partners

**Figure 6:**
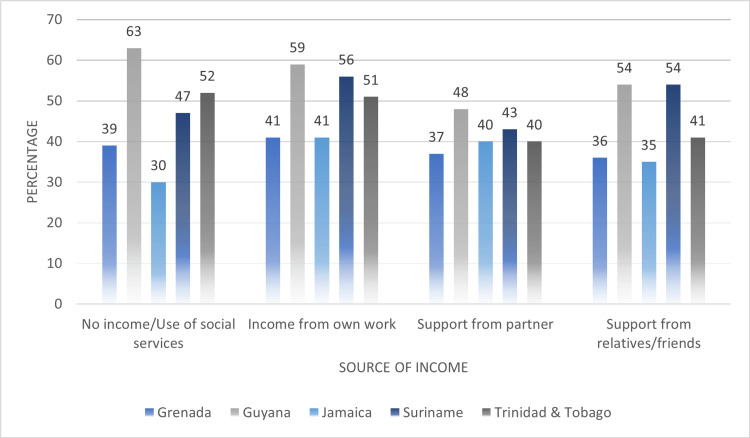
Comparing sources of income among women who experienced any type of intimate partner violence (IPV) across their lifetime

In Figure 6, we compared sources of income among women who were subjected to any type of IPV. It was observed that, on average, almost 50% of women who earned their own income experienced any type of IPV in their lifetime (no income: 46.2%, income from work: 49.6%, support from partner: 41.6%, support from family and friends: 44%). This average point prevalence was closely followed by women who did not earn an income and those who depended on financial support from family and friends. Guyana presented the highest point prevalence of IPV in women who did not earn an income (63%). Overall, Guyana (59%) and Suriname (56%) reported the highest proportions of women experiencing IPV given that they earned their own income. This was also the case among women who relied on financial support from family and friends (Guyana: 54%, Suriname: 54%).

**Figure 7:**
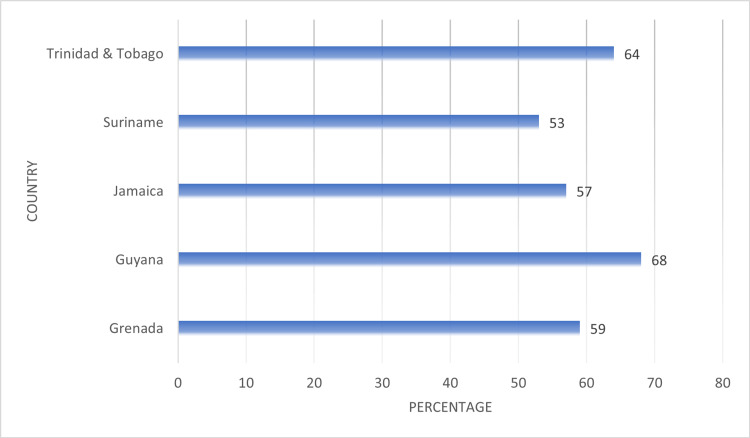
Comparing reports of controlling behaviors by partners among women who experienced intimate partner violence (IPV) across their lifetime

In Figure 7, we compared controlling behaviors of partners reported by women who were subjected to any type of IPV. On average, 60.2% of women who experienced IPV in their lifetime reported accounts of their partners exhibiting controlling behaviors. Guyana (68%) and Trinidad and Tobago (64%) displayed the highest proportions for this factor.

Institutional factors of the Social-Ecological Framework: country comparisons of disclosure of violence, health-seeking behaviors

**Figure 8:**
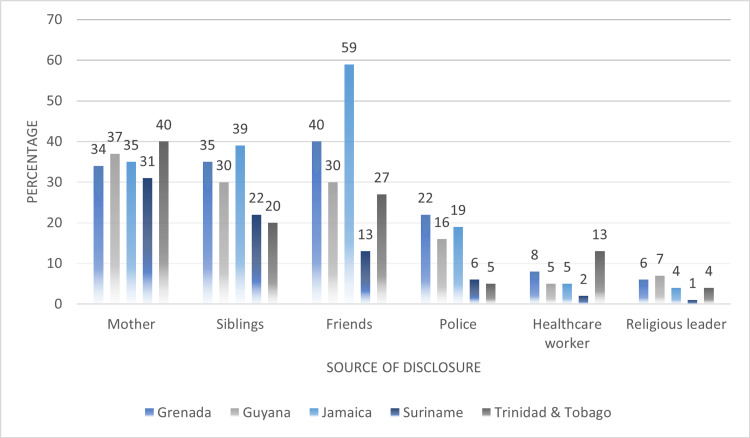
Comparing sources of disclosure of violence among women who experienced any type of intimate partner violence (IPV) across their lifetime

In Figure 8, we compared sources of disclosure of violence among women who were subjected to any type of IPV. On average, the highest proportions of women experiencing IPV disclosed these ordeals to their mothers or friends (mother: 35.4%, siblings: 29.2%, friends: 33.8%, police: 13.6%, healthcare worker: 6.6%, religious leader: 4.4%). Trinidad and Tobago reported the highest proportions of women experiencing IPV and disclosed this to their mothers (40%), while Jamaica had the highest proportions disclosing their experience to siblings (39%) and friends (59%).

**Figure 9:**
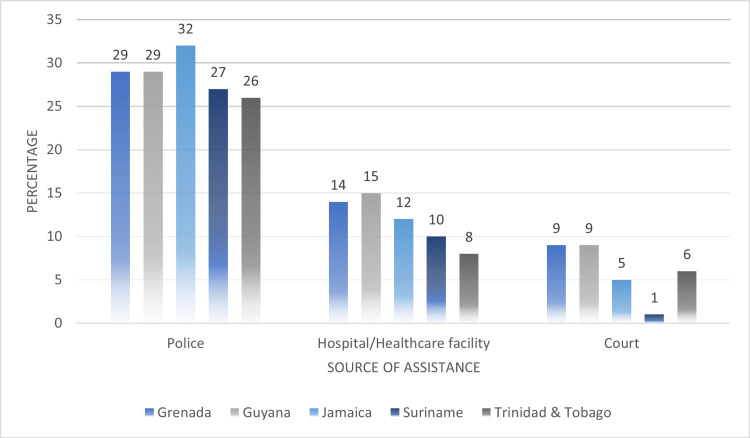
Comparing help-seeking behaviors among women who experienced any type of intimate partner violence (IPV)

In Figure 9, we compared help-seeking among women who were subjected to any type of IPV. The highest average proportion of women who experienced IPV reached out to the police for assistance (police: 28.6%, hospital or another healthcare facility: 11.8%, court: 6%).

Societal factors of the Social-Ecological Framework: country comparisons of the age differences of partners, age of first sexual experience

**Figure 10:**
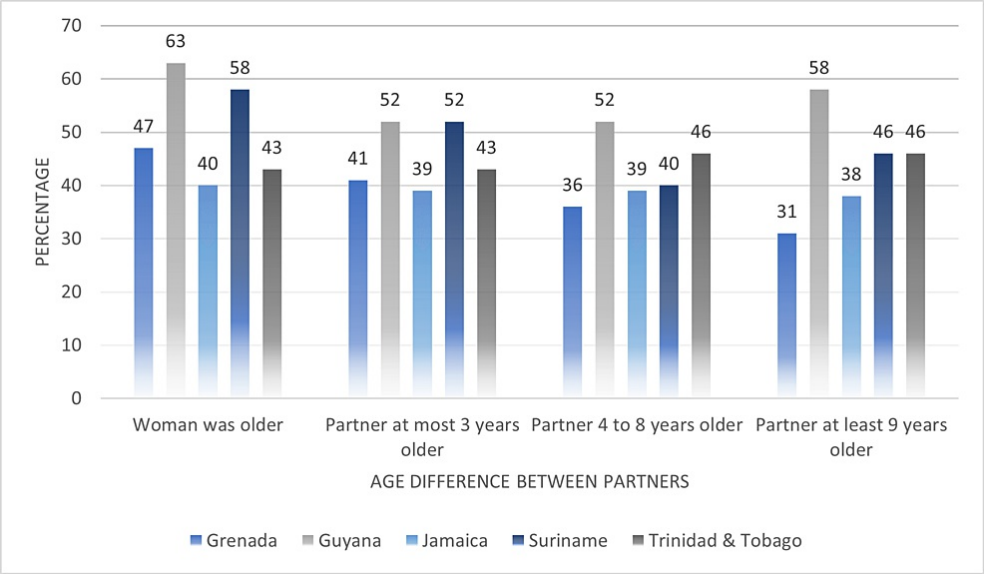
Comparing the age differences of partners among women who experienced any type of intimate partner violence (IPV)

In Figure 10, we compared the age differences of partners among women who were subjected to any type of IPV. The average point prevalence of IPV among women was highest in relationships where the woman was older (woman was older: 50.2%, partner at most three years older: 45.4%, partner four to eight years older: 42.6%, partner at least nine years older: 43.8%). Guyana reported the highest proportions of IPV among women given that the woman was older in the relationship (63%). This was closely followed by Suriname (58%). Guyana and Suriname also reported a 52% point prevalence of IPV among women given that their partner was at most three years older. For women in relationships with a partner who was four to eight years or at least nine years older, the proportions experiencing IPV were over 50%.

**Figure 11:**
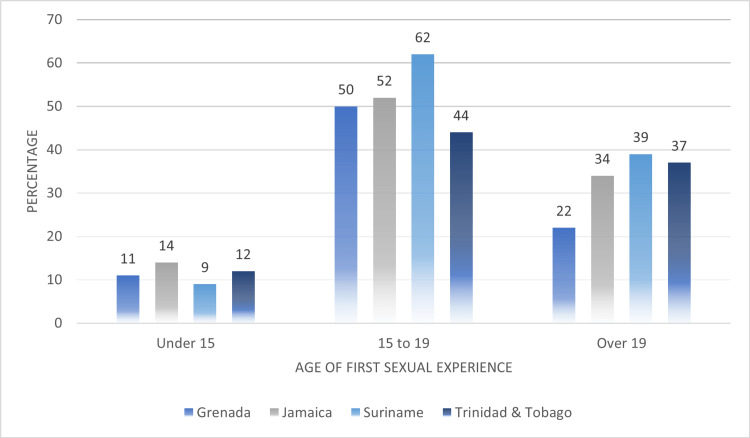
Comparing the age of first sexual experience among women who experienced any type of intimate partner violence (IPV)

In Figure 11, we compared the ages of first sexual experiences among women who were subjected to any type of IPV. Data from Guyana was not available for this factor. However, the highest average proportion of women who experienced some form of IPV had their first sexual experience between the ages of 15 and 19 years (under 15 years: 11.5%, 15 to 19 years: 52%, over 19 years: 33%). Grenada (50%), Jamaica (52%) and Suriname (62%) reported proportions of women who experienced IPV of at least 50%.

## Discussion

Guided by the Social-Ecological Framework, the results of our comparative analysis across five countries emphasized factors at the individual, interpersonal, institutional, and societal levels associated with women risks for IPV. In this section, we discussed the results in the context of women’s dual risk for HIV infection across each level of the Social-Ecological Framework. We also discussed the applicability of the Framework for combined violence and HIV prevention interventions at the individual, interpersonal, institutional and societal level.

The Caribbean have been reported to have the second highest HIV prevalence outside of Southern Africa. In 2020, the UNAIDS estimated that 330,000 persons to be living with HIV infection in the Caribbean. The HIV epidemic has been both generalized and concentrated among subpopulations such as men who have sex with men (MSM), sex workers and other key population groups. Among countries in the Caribbean, Haiti was reported to have the highest number of people living with HIV, followed by the Dominican Republic and Cuba [15]. While the number of new infections has declined between 2010 and 2020, women in general (15-49 years) overlapping with young adults (15-24 years), account for a higher proportion of new HIV cases, suggesting a disproportionately higher rate of infection among younger women in the Caribbean.

Social-Ecological Framework: individual factors

At the individual level of the Social-Ecological Framework, our analysis showed individual factors such as younger age, lower education level, and having a history of childhood abuse were associated with women’s risk for IPV across the five counties studied. These results were consistent with the findings globally, and in the context of women’s risk for HIV infection.

The results of our analysis showed that on average approximately 34% women subjected to IPV were in the age groups 15 to 24 years and 25 to 34 years suggesting a higher point prevalence of among younger women. On average of 50% of women reported having primary school education or lower and 63% of women reported some form of childhood abuse. In the context of HIV epidemic in the region, women in general (15-49 years) overlapping with young adults (15-24 years), account for a higher proportion of new HIV cases, suggesting a disproportionately higher rate of infection among younger women. Studies have also shown that women with lower educational attainment may be less knowledgeable about risks and therefore less able to adopt HIV risk-reducing behaviors. While limited research has been published on the association between childhood sexual abuse and HIV transmission risks in the Caribbean, results from a systemic review showed that sexual risk behaviors were almost twice times more likely among adult women who experience childhood sexual abuse compared to their counterparts, hence putting them at higher risk for sexually transmitted infections [16]. A cross-sectional study conducted in Haiti analyzing gender differences in the associations between childhood sexual abuse and substance use and sexual risk behaviors found an increased drug use among women who experienced childhood sexual abuse [17]. Given the presence of co-occurring risk factors for intimate partner violence and HIV, stakeholders across government agencies can plan and design target interventions focusing on younger women, increasing access to educational opportunities and appropriate peer counselling at schools, health care settings, youth centers etc., to promote early detection and reduce risk for intimate violence and HIV among young women.

Social-Ecological Framework: interpersonal factors

At the interpersonal level of the Social-Ecological Framework, the results of our analysis showed factors such as alcohol use by partners, income dependency, and controlling behaviors of partners were associated with women’s risk of IPV across the five countries. On average, around 60% of women subjected to any type of IPV in their lifetime reported alcohol use by their partners at least once per week. Our results were also consistent with global findings as the use of alcohol and IPV have been shown to have a significant relationship in a systemic review and meta-analyses study [18]. The comparison of sources of income reported by women subjected to any type of violence showed that on average 46% of women reported having no source of income, and an additional 42% indicated they relied on the income of their partners. The results also showed that on average 60% women subjected to any type of IPV reported at least one controlling behavior by their partner and or spouse.

The existing research on the behaviors that increases women’s the risk for HIV infection also underscores the association with interpersonal risk factors such as alcohol use, dependency on the income from partners and by extension the controlling behaviors of partners. The use of alcohol and drugs have been reported to contribute directly and indirectly to increasing risk for HIV acquisition for both male perpetrators as well as women who experience intimate partner violence. While data is limited in the Caribbean, traditional gender roles still exist in many countries imply that women should be submissive, allowing men to make decisions about engaging in sex. Studies outside of the region found that alcohol contributes to casual sex, acts as a disincentive to condom use, and increases the risk of HIV infection [19]. Past studies have also shown that women who experience intimate partner violence and sexual abuse are more likely are more likely to misuse alcohol, and less likely to have control over sexual decisions, further putting them at increased risk for HIV infection [20,21]. Women who are dependent on their partners and/or spouses may have little choice but to adopt behaviors that put them at risk of infection, including transactional and intergenerational sex, and relationships that exposed them to violence and abuse [22]. These co-occurring interpersonal level risk factors underscore the applicability of the Social-Ecological Framework in identifying multiple drivers of risk for both intimate partner violence and HIV infection and further assist to design and implement integrated interventions. At the interpersonal level, it would be beneficial to program planners to develop opportunities for employment and skills training for women, promote partner counselling in the area of conflict resolution and healthy relationships, increasing access to marriage and counselling therapy for couples and referral to corrective programs to address alcohol use.

Social-Ecological Framework: institutional factors

The results of our analysis found that institutional factors of the Social-Ecological Framework namely, disclosure of violence in the context of seeking medical care and as a result deterring the use of health services were associated with IPV. On average over 30% of women reported disclosing violence to either to their network of family or friends and approximately 7% disclosed to public health centers and hospitals. These suggest that women may deter seeking much-needed health services, representing a missed opportunity to address the physical and mental health consequences as a result of experiencing intimate partner violence. In the context of women’s risk of HIV, research has shown that women’s exposure to violence is associated with long-term health consequences, including high rates of depression, gastrointestinal symptoms, neurological disorders, reproductive health problems and increased risks of sexually transmitted infections [23]. In addition, it has been shown that women disclosing psychological abuse were two times more likely to experience an STI. Given these associations, the Social-Ecological Framework is also applicable to understanding the drivers of intimate partner violence and risk for HIV infection at the institutional levels. Further, the Framework can assist to design integrated interventions to address the barriers to seeking health services for women experience violence and those at increased risk for HIV infection. Healthcare institutions should promote a non-stigmatizing and trauma sensitive environment that enhances rather than discourages the identification of abuse and educates clients about their risk for HIV infection [24].

Social-Ecological Framework: societal factors

In our study, societal norms fueled by gender inequalities was found to be consistently associated with the experience of IPV among women in the Caribbean. Among the countries surveyed, we found that societal norms such as the age differences between partners, and age of first sexual experiences to be associated with IPV. In a 2021, the UNAIDS reported that on average 9% to 24% of young women in the Caribbean reported having sex with a man at least 10 years older than themselves. The results of our analysis confirm the UNAIDS report, as we found that on average that 44% % of women experiencing lifetime IPV reported an age difference of nine years or older between themselves and their spouses/partners. Additionally, 64% of women surveyed reported their first sexual experience at the age of 19 or younger. 

In the context of HIV, gender inequalities in the form of harmful social norms reinforce unequal power dynamics between men and women reducing the ability of women and girls to negotiate condom use, impacting their sexual and reproductive health rights, and decision making in matters regarding their relationships [25]. These norms place women and girls at a disadvantage, increasing their vulnerability and risks for HIV infection. Globally women living with HIV also face stigma, which is also aggravated by their lack of rights and IPV experiences [26]. While countries in Latin America and Caribbean have made some progress in closing the gaps in gender inequalities, the Global Gender Gap report (2023) indicated it will take the region 53 years to achieve gender parity, further reinforcing the pervasiveness of social norms and ideologies and its multi-dimensional impact on women's health and survival, economic participation and opportunities, educational attainment, and political empowerment [27]. Gender inequalities are also pervasive at the institutional level of the Social-Ecological Framework, and reflective of the social inequalities that impact women access to care

In this regard, the Social-Ecological Framework is applicable to understanding of gender inequality as both a root cause and driver of IPV and HIV infection among women and girls and others affected by the dual epidemic. The use of the Social-Ecological Framework is also noteworthy as a predictive model and in advocating for systemic changes to diminish/reverse the effects of harmful social norms, protect women rights against violence, and promote policies to reduce structural inequalities and discriminatory gender practices.

Study limitations

This paper focused exclusively on IPV i.e., the most common forms of violence against women, and a review of the co-occurring risk factors contributing to their IPV and HIV risk of infection. In the absence of more robust data for the Caribbean, the aim was to conceptually use the Social-Ecological Framework to link the drivers of the dual epidemic in our region. 

The study and data is limited as it did not discuss or incorporate sexual abuse, survivors of sex trafficking and other forms of exploitation against women and how these increases their risk for HIV infection. Further, their are subpopulations who as a result of their sexual orientation and gender identify are at increased risk for Intimate partner and other forms of violence. Also given the available data, the associated pathways linking gender inequality to women's risks for IPV and HIV infection were limited. 

## Conclusions

The dual burden of gender-based violence and HIV infection presents an ongoing public health threat across countries globally. For the Caribbean i.e., a region reported to have the second highest prevalence of HIV, intimate partner violence can increase the chances of acquiring HIV among women. 

In the context of our findings, our study underscores the use of the Social-Ecological Framework as an analytical tool helping to strengthen our understanding of multilevel factors as drivers of IPV and HIV infection risks occurring at the same time among women in our region. Secondly, given these multilevel and co-occurring risks, our study recommends the use of the Social-Ecological Framework as a predictive tool assisting to plan prevention interventions at the individual, interpersonal, institutional and societal levels toward the aim of reducing women's dual risks for IPV and HIV infection in our region. Thirdly, the Social-Ecological Framework is recommended as an approach to strengthen behavior change communication and the coordination of prevention efforts across multiple sectors advocating for change, and the modification of risks at the individual, interpersonal, institutional and societal levels. Future studies can build on use of the Social-Ecological Framework to explore the drivers of risk among key population groups such as men who have sex with men, also noted to be disproportionally affected by HIV and IPV in our region.

To address the gaps in our study, a concerted effort must be made to strengthen data and research to better characterize the prevalence of IPV and HIV infection and the associated risk factors among women and girls and other affected populations in the Caribbean. Additionally, comprehensive data linking social, economic, educational, and political indicators are needed to increase our understanding of the effects of harmful social norms and to address gender inequalities that fuel IPV and HIV infection rates among women in our region and globally. 
